# *N*-Glycan Isomer Differentiation by Zero Flow Capillary Electrophoresis Coupled to Mass Spectrometry

**DOI:** 10.1021/acs.analchem.2c02840

**Published:** 2022-09-13

**Authors:** Sander Wagt, Noortje de Haan, Wenjun Wang, Tao Zhang, Manfred Wuhrer, Guinevere S. M. Lageveen-Kammeijer

**Affiliations:** Leiden University Medical Center, Center for Proteomics and Metabolomics, 2300 RC Leiden, The Netherlands

## Abstract

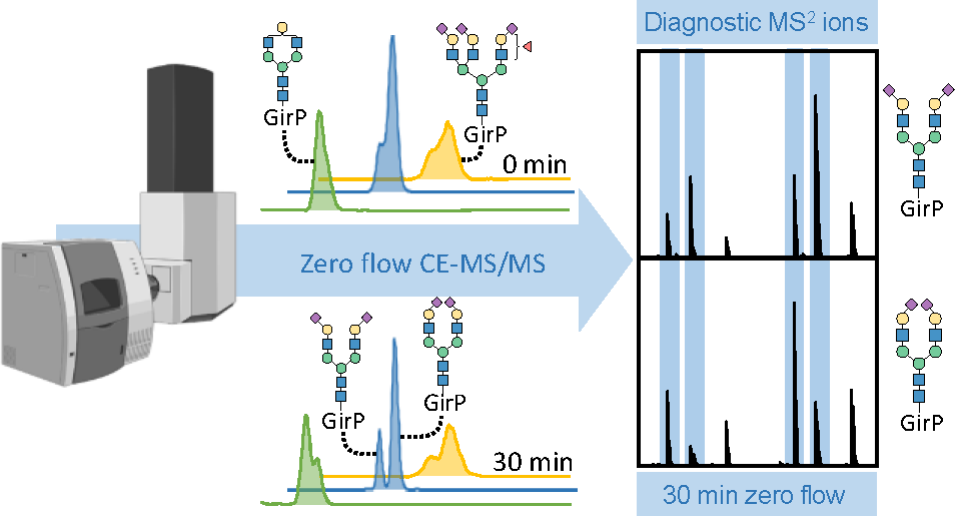

Isomeric *N*-glycans often vastly differ in their biological activities, hence the need for methods that allow resolving and structurally characterizing them in biological material. Here, we established a zero flow approach using capillary electrophoresis in combination with (tandem) mass spectrometry to allow structural characterization of isomeric *N*-glycans at high sensitivity. Additionally, diagnostic fragment ion ratios were identified, indicative for the antenna carrying specifically linked sialic acids. In total, 208 *N*-glycans were characterized in human plasma, with 57 compositions showing multiple isomers.

## Introduction

Glycosylation is one of the most frequently occurring post-translational protein modifications, and the thorough investigation of this modification has led to the widespread recognition that aberrant glycosylation patterns are correlated with various diseases and their progression.^1,2^ To decipher glycan structure–function relationships, an in-depth characterization of glycan structures is essential.^2^ A major analytical challenge lies in the full structural characterization of isomers, such as positional and linkage isomers, which display distinct functional epitopes.^3,4^ For the separation of isomers, several analytical strategies have been established, which are mainly based on liquid chromatography (LC) (e.g., porous graphitized carbon (PGC), reversed-phase (RP), and hydrophilic-interaction (HILIC)), along with complementary approaches founded on capillary electrophoresis (CE) and complemented by ion mobility-mass spectrometry (IM-MS).^5−9^

Among these, CE has extensively been used for several decades, primarily in combination with laser-induced fluorescence detection.^10^ More recently, the focus has shifted toward the hyphenation of CE to MS via electrospray ionization (ESI), supported by major technological advances in the field, including commercialization of a sheathless CE-MS platform for high sensitivity.^9^ CE-ESI-MS is an attractive analytical platform in many areas, given its highly efficient separation mechanism, high sensitivity, and low sample consumption.^11,12^ We previously illustrated the power of this platform in glycomics, obtaining in-depth released *N*-glycan profiles from biological material, reaching a dynamic range of 4 orders of magnitude and a detection limit at the low attomole level.^13^

In the current study, we built on our previous approach and developed a new method to allow extensive glycan isomer separation, while maintaining the sensitivity of the original CE-ESI-MS glycomics platform. This was achieved by using a statically coated neutral capillary which diminishes the electro-osmotic flow (EOF),^14,15^ thereby enabling so-called “zero flow” CE (i.e., separation is only achieved based on the electrophoretic mobilities of the analytes by solely applying a separation voltage and without applied pressure; Table S1).^15^ Using the new methodology, isoforms were observed for many glycan compositions, including different antenna configurations and decorations such as sialic acid linkage isomers.

## Experimental Section

### Chemicals

Ultratrol dynamic precoat LN was obtained from Target Discovery (Palo Alto, CA). Glacial acetic acid (HAc; cat. no. 15625660), hydrochloric acid solutions of 1 M (HCl; cat. no. 15653530), and 7.5 M ammonium acetate (AmAc; cat. no. A2706) were purchased from Sigma-Aldrich (Steinheim, Germany). H3N4, H5N4, and H7N6 (CN-NGA2-10U, CN-NA2-10U, CN-NA4-10U, respectively) were kindly provided by Ludger Ltd. (Abingdon, U.K.). Acetonitrile (MeCN; cat. no. 1203502) was obtained from Biosolve (Valkenswaard, The Netherlands). LC-MS grade water (cat. no. 15641400) was obtained from Riedel-de Haën (Buchs, Switzerland).

### *N*-Glycan Sample Preparation for CE-ESI-MS Measurements

The *N-*glycan sample was prepared according to the recently described workflow with some brief modifications.^13^ Detailed information about the workflow is provided in the Experimental Section in the Supporting Information.

### CE-ESI-MS(/MS) Analysis

All experimental analyses were conducted on a CESI 8000 system (SCIEX, Framingham, MA), coupled to a UHR-QqTOF maXis Impact HD mass spectrometer (Bruker Daltonics, Bremen, Germany) through the OptiMS Bruker MS adapter (SCIEX) modified for the use of a dopant enriched nitrogen (DEN)-gas supply^21^ (nanoBooster technology from Bruker Daltonics).

The CE separations were performed on OptiMS cartridges (91 cm long, 30 μm i.d., 150 μm o.d.; SCIEX) with two types of neutrally coated capillaries: (1) generated using an in-house developed procedure with Ultratrol dynamic precoat LN^13^ or (2) generated with the commercially available neutral OptiMS cartridge (SCIEX). Separation was conducted at 20 °C using a background electrolyte (BGE) consisting of 10% HAc (pH 2.3). The samples were dissolved in 2.5 μL of leading electrolyte (final concentration of 100 mM AmAc, pH 4.0) and loaded onto the capillary by a hydrodynamic injection of 1 psi for 60 s (8.7 nL/1.35% capillary volume). To identify the two H5N4S_2,3_1S_2,6_1 isomers, released *N*-glycans from fetuin were spiked into the labeled TPNG sample by different ratios of 0:1, 1:1, and 2:1 on CE-ESI-MS measurement (*N* = 2). After applying 2.5 psi for 10 s to add a BGE post plug, online preconcentration of the sample was obtained by transient-isotachophoresis (t-ITP).^14,15^

The mass spectrometer (MS) was operated in positive ion mode, and electrospray ionization (ESI) was achieved using a capillary voltage of −1300 V and DEN-gas with MeCN as dopant at 0.2 bar. The temperature and drying gas (nitrogen) flow rate were set at 150 °C and 1.2 L/min, respectively. MS as well as tandem MS (MS/MS) spectra were acquired between *m*/*z* 200 and 2000, using 1 Hz as the spectral acquisition rate.

From each MS spectrum, the three most abundant precursor ions observed in the 2^+^ or 3^+^ charge states were fragmented when present at an intensity of at least 4548; fragmentation of each precursor could occur three times before being excluded for 48 s. Depending on the *m*/*z* value, MS/MS spectra were acquired using an isolation width of 8–10 Da. In a similar manner, this *m*/*z* dependency was also used to set the collision energies as a linear curve for all charge states, which started from 55 eV at *m*/*z* 700 up to 124 eV at *m*/*z* 1800, to which a basic stepping mode was applied by operating at 100% or 50% of the collision energies for respectively 80% or 20% of the time.

### Dynamically Coated Neutral Capillary

The dynamically coated neutral capillary, OptiMS silica surface cartridges (SCIEX) were coated with UT according to the recently described procedure (for more details, see the Experimental Section in the Supporting Information).^13^

### Statically Coated Neutral Capillary

The commercially available neutral OptiMS cartridges containing a static neutral coating, capillaries, were conditioned by submerging the porous capillary tip into H_2_O and consecutively flushing the forward line with 0.1 M HCl (5 min) and 50 mM AmAc pH 3.0 (10 min) at 100 psi. Then, H_2_O was used to flush the conductive liquid line (5 min, 75 psi) and separation line (30 min, 100 psi). Following the conditioning steps, the capillary coating was rehydrated by flushing it with H_2_O (16–18 h, 10 psi). Prior to each run, the capillary was flushed with 0.1 M HCl (5 min, 100 psi), followed by filling the forward (10 min, 100 psi) and reverse (3 min, 75 psi) line with BGE. Separation was conducted at 30 kV and 0.5 psi. The pressure was applied to both the forward and reverse lines and was either applied directly from the start of the analysis or after waiting various periods of up to 30 min. A delayed application of the pressure resulted in a period of zero flow separation, exclusively driven by the electrophoretic mobility.

### CE-ESI-MS Data Processing

Data analysis was performed using the raw CE-ESI-MS data by DataAnalysis 5.0 (Build 203, Bruker Daltonics). Raw CE-ESI-MS data were calibrated prior to data analysis using a minimum of five signals of the identified *N*-glycan compositions (Table S2). Extracted ion electropherograms (smoothed with a Gaussian fit) were acquired for *N*-glycan compositions previously observed in human plasma, including the different derivatized sialic acid linkage isomers,^13^ by extracting the first three isotopes of the singly, doubly, triply, and quaternary charged analytes if *m*/*z* > 650 and using an *m*/*z* window of ±0.02 Th. The EIEs were then used to determine the migration time of the analytes, for which the accurate mass (±20 ppm) and the isotopic peak pattern (comparison to the theoretical isotopic pattern by eye) were evaluated.

Subsequently, the full list of all assigned peaks was used to process the data of the MS/MS analysis. Here, we first screened each peak if a matching fragmentation spectrum could be found, which was then used to elucidate the corresponding *N*-glycan composition based on the diagnostic B- and Y-ions obtained by glycosidic bond cleavage. To this end, the Y-ions were used to sequence the *N*-glycan structure starting from the GlcNAc to which the label is attached. The initial starting point is depending on whether there is a core fucose present (*m*/*z* 501.220) or not (*m*/*z* 355.162). The B-ions were primarily used to identify sialylated *N*-glycans by a trisaccharide fragment (*N*-acetylhexosamine + hexose + sialic acid), resulting in *m*/*z* 656.251 for the α2,3-linked and *m*/*z* 685.260 for the α2,6-linked sialic acid.

For the PGC-nano-LC-MS sample preparation, analysis, and data processing, see the Experimental Section in the Supporting Information.

## Results and Discussion

**Figure 1:**
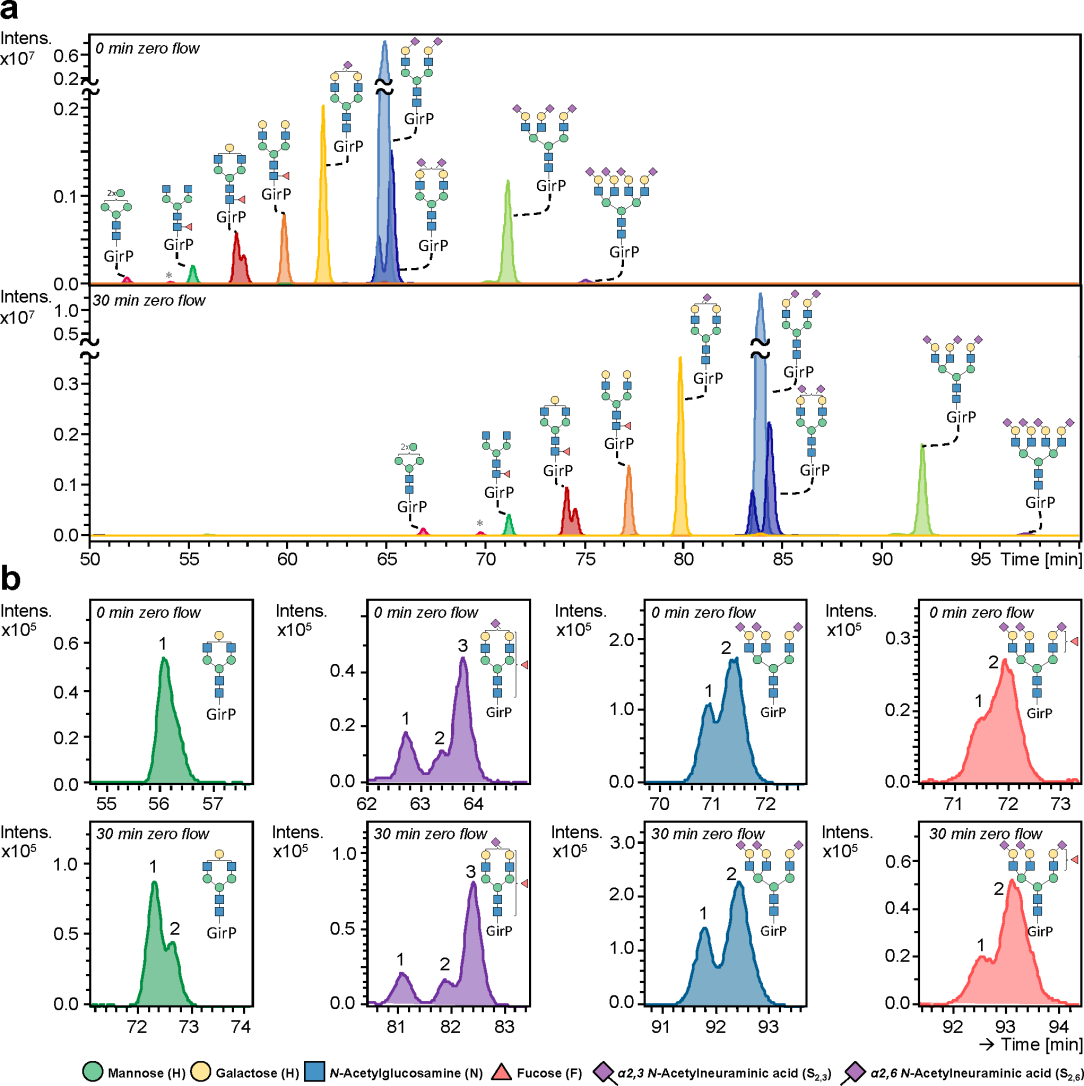
CE-ESI-MS analysis of *N*-glycans released from human plasma after differential sialic acid derivatization and cationic reducing end labeling. (a) Extracted ion electropherograms (EIEs) from nine of the most abundant *N*-glycans observed across the entire migration window, separated with the commercially available, statically coated neutral capillary (OptiMS, SCIEX). Significant differences in the separation efficiency are obtained by applying a period of 0 min (top) and 30 min (bottom) zero flow. (b) Magnification of EIEs of H4N4, H5N4F1S_2,3_1, H6N5S_2,3_2S_2,6_1, and H6N5F1S_2,3_2S_2,6_1, in which the number of isomers is depicted, together revealing the resolving power obtained by applying 30 min zero flow. * Indicates an in-source decay product of the H6N2. The depicted structures are based on exact mass, literature, and knowledge of the biosynthetic pathway; monosaccharide linkages were not determined except for the sialic acids.

*N*-Glycan isomer separation by CE-MS was achieved while high sensitivity, being a performance feature of the previously established method,^13^ was maintained. We prepared released *N*-glycans for their analysis by differential sialic acid derivatization and performed a uniform positively charged reducing end tagging of all glycans. In the optimization of the CE method, we succeeded in vastly suppressing the EOF and improving the separation by replacing the dynamically Ultratrol LN (UT) coated capillary for a commercially available neutral capillary with static coating (Figure S1). To maintain ESI, a constant hydrodynamic pressure (0.5 psi) was applied, resulting in a minimal flow of approximately 4 nL/min. This resulted in an improved *N*-glycan separation window that increased from 12 min (UT) up to 24 min (static) and featured improved isomer resolution, as exemplified by the different migration behavior of the isomers with the compositions H5N4S_2,3_1S_2,6_1 and H6N5S_2,3_1S_2,6_2 (Figure S1). To further increase the separation of isomeric *N*-glycans, we employed a zero flow approach that consists of two steps.^15^ In the first step, the separation power of the CE was improved by performing a virtually flowless CE (in the absence of EOF and hydrodynamic flow). This step eliminates ESI and (tandem) MS detection and was regained in the second step, by generating a stable hydrodynamic flow of approximately 4 nL/min (0.5 psi). The zero flow regime allowed the *N*-glycan separation window to be broadened from 24 to 31 min using 30 min zero flow (Figure 1a). Importantly, an increase in isomer resolution was obtained for many compositions, exemplified by—but not limited to—H4N4, H5N4F1S_2,3_1, H6N5S_2,3_2S_2,6_1, and H6N5F1S_2,3_2S_2,6_1 (Figure 1b). Of note, the implementation of zero flow did not skew glycan quantification, as demonstrated for 30 common *N*-glycan compositions found in the total human plasma *N*-glycome (TPNG) (Figure S2).

**Figure 2:**
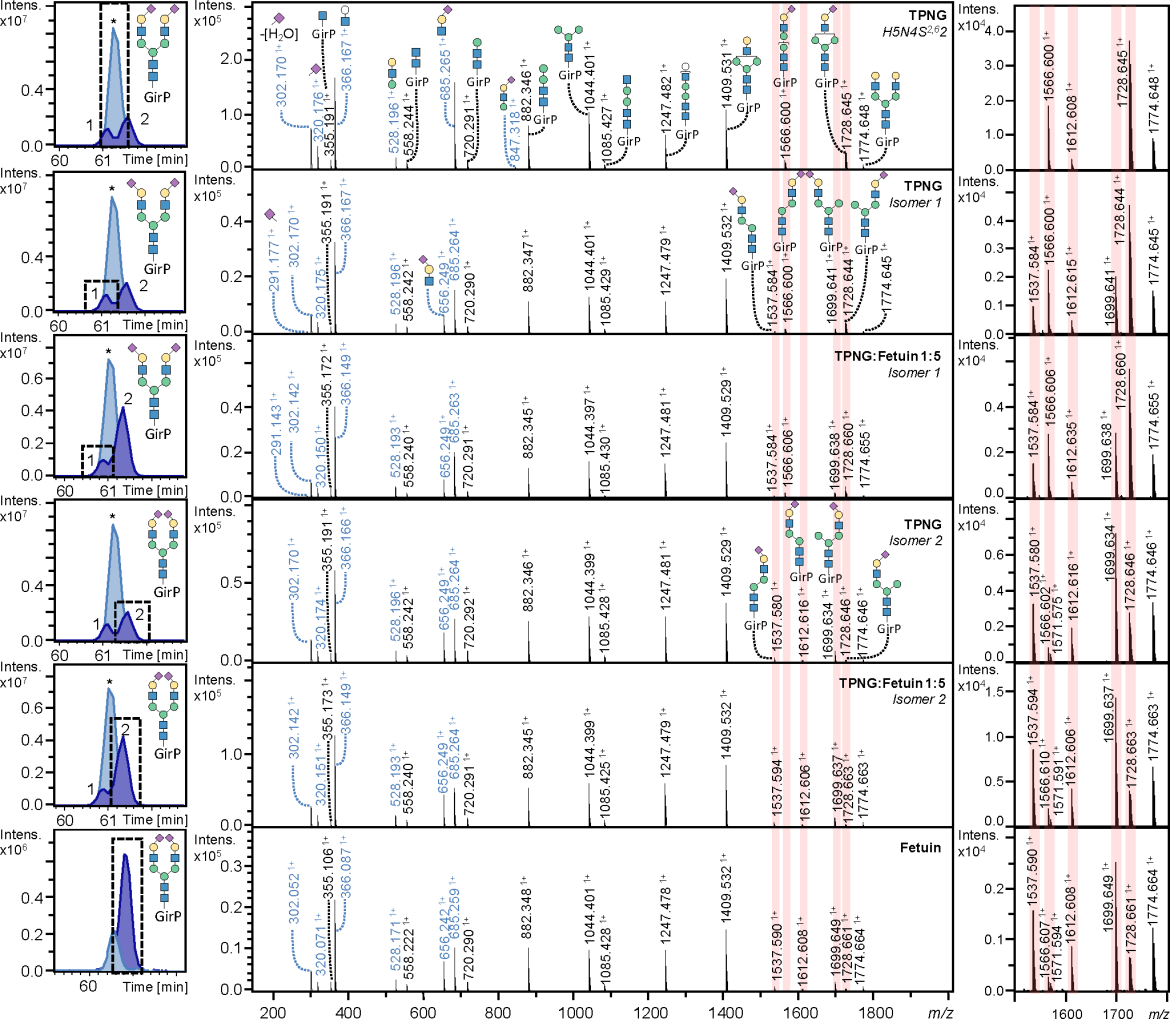
CE separation and structural elucidation of isomeric *N*-glycans with the composition H5N4S2 after differential sialic acid derivatization and cationic reducing end labeling. Various samples were analyzed including released *N*-glycans from TPNG, TPNG spiked with fetuin in a 1:5 ratio (TPNG:fetuin 1:5), and fetuin. (left) Extracted ion electropherograms of the masses belonging to *N*-glycans H5N4S_2,6_2 (light blue and marked with *) and H5N4S_2,3_1S_2,6_1 (dark blue) in the various samples. (middle) MS/MS fragmentation spectra obtained from each of the (left) separated peaks in which the B- and Y-fragment ions are labeled in blue and black, respectively. (right) Magnification of the MS/MS fragmentation in *m*/*z* 1500–1800. Highlighted in red are the Y-ions of diagnostic value. The legend is the same as that in Figure 1.

Structural characterization of the isomers was supported by MS/MS using collision induced dissociation (CID), as exemplified for the H5N4S2 species H5N4S_2,3_1S_2,6_1 and H5N4S_2,6_2 (Figure 2). Close to baseline separation was obtained for the H5N4S_2,3_1S_2,6_1 isomers when a 30 min zero flow period was applied. The different sialic acid linkages could be distinguished based on their mass due to the differential sialic acid derivatization. MS/MS confirmed the presence of both an α2,3-sialylated and α2,6-sialylated antenna, showing B-ions at *m*/*z* 656.251 and *m*/*z* 685.260, respectively. Interestingly, the fragmentation spectra obtained from the two separated H5N4S_2,3_1S_2,6_1 isomers (Figure 2, right) showed pronounced differences between the ratios of the Y-ions at *m*/*z* 1728.663 (H4N3S_2,6_1) and 1699.641 (H4N3S_2,3_1), as well as *m*/*z* 1566.617 (H3N3S_2,6_1) and 1537.607 (H3N3S_2,3_1). This suggests a more facile loss of the 6′-linked or 3′-linked antenna upon fragmentation, independent of the sialic acid linkage. To investigate this effect on fragmentation further, the *N*-glycome of fetuin was analyzed, for which the exact structure of the most abundant H5N4S_2,3_1S_2,6_1 isomer has been determined by both PGC-LC-MS^16^ and NMR analysis.^17^ This structure carries the α2,6-sialylated antenna on the 3′-linked mannose and the α2,3-sialylated antenna on the 6′-linked mannose. To assign one of the TPNG-derived isomeric peaks to this structure, fetuin *N*-glycans were spiked into the released TPNG *N*-glycome sample in various amounts. As the second migrating TPNG isomer co-migrated with the fetuin glycan and showed the same diagnostic ion ratios in MS/MS, we concluded that the second isomer has the α2,6-sialylated antenna on the 3′-linked mannose, while the 6′-linked mannose carries the α2,3-sialylated antenna. The first-migrating isomer was assumed to exhibit the reversed antenna sialylation pattern (α2,6-sialylation on the 6′-arm and α2,3-sialylation on the 3′-arm) as it seemed unlikely that the isomer separation is based on other structural features, especially as no such heterogeneity was observed for the uniformly α2,6-sialylated *N*-glycan. This indicates that the cleavage of the Man-α1,3-Man bond as well as of the GlcNAc-β1,2-Man bond on that antenna is, under the current derivatization regime, more facile than cleavages of the 6′-branch, independent of sialic acid linkage. Knowledge of the stability of differently linked monosaccharides and their effect on fragmentation patterns is limited. A previous study by Lane et al. showed that a fully α2,3-sialylated *N*-glycan exhibits a different fragmentation pattern than its fully α2,6-sialylated equivalent using differential mobility spectrometry and negative mode CID on native glycans, but it did not investigate the fragmentation patterns of isomeric species of H5N4S_2,3_1S_2,6_1.^18^ The Mechref lab observed isomeric separation of the permethylated *N*-glycan H4N4F1 using PGC-LC-MS and found diagnostic ion ratios dependent on the galactose branching.^19^ They demonstrated that a galactosylated 6′-arm was better stabilized than a galactosylated 3′-arm. Although this stabilizing effect was attributed to the presence of a core fucose, this is in line with our findings on non-fucosylated species. It should be noted that the stability of the glycans and the resulting fragmentation pattern will be affected by the derivatization and applied fragmentation polarity. Further investigation of diagnostic ion ratios after sialic acid derivatization and using positive mode CID will greatly help the future annotation of isomeric species.

While PGC-LC-MS is the state-of-the-art method for glycan isomer separation, we here show that CE-ESI-MS is a competitive alternative. Performing a comparative analysis of the sensitivity of the two methods, we showed that our CE-ESI-MS platform outperforms the PGC-LC-MS one with a factor of 1000. We determined the limit-of-detection for the PGC-LC-MS platform (negative ionization mode) in the fmol range (Table S2), while the CE-ESI-MS platform showed a limit-of-detection in the low amol range.^13^

To further demonstrate the performance of the zero flow method, the *N*-glycome of human plasma was analyzed. In this complex biological matrix, a total of 208 *N*-glycans were identified, of which 135 had a unique composition (including the differentiation between α2,3- and α2,6-linked sialic acids as assigned based on their derivatized mass). From these 135 compositions, 57 *N-*glycan compositions showed the presence of isomeric species, which accounted for the additional 73 *N*-glycans observed (Table S3). The isomers showed variation in antenna configuration and decoration. While not all isomers found in the current study were structurally assigned, the differential fragmentation patterns as described above for the diantennary *N*-glycan were observed for various *N*-glycan isomers (Figure S3), and the use of these characteristic Y-ion patterns for glycan structural assignment warrants further investigation.

## Conclusion

In this study, we presented a method that achieves *N*-glycan isomer differentiation by a combination of sialic acid derivatization, zero flow CE separation, and tandem MS. As such, the method provides both the high sensitivity as well as the high resolution and isomer differentiation needed to address pressing glycobiological questions in biomedicine. We envision that the method will facilitate the in-depth glycomic analysis of minute amounts of biofluids and cellular or tissue material. Additionally, we think that the high-resolution zero flow CE-ESI-MS approach is not only valuable for the glycomics field but could also be utilized in metabolomic, lipidomic, and proteomic studies where sample amounts are restricted and resolution as well as isomer differentiation is essential for achieving analytical depth and biological insights.

## Supporting Information Available

**Experimental Section,** chemicals, *N*-glycan sample preparation for CE-ESI-MS and PGC-nano-LC-MS measurements, dynamically coated neutral capillary, PGC-nano-LC-MS/MS analysis, and data processing; **Figure S1,** CE-ESI-MS analysis of released and positively labeled *N*-glycans on different neutral capillaries compared based on the separation efficiency; **Figure S2,** relative peak areas obtained from the CE-ESI-MS analysis of derivatized and positively labeled *N*-glycans released from human plasma on a static neutrally coated capillary; **Figure S3,** CE separation and structural elucidation of isomeric *N*-glycans with the composition H6N5S3 after differential sialic acid derivatization and positive reducing end labeling; **Table S1,** schematic overview of method properties per capillary type; **Table S2,** sensitivity assessment (*N* = 3) using two glycan standards (H3N4 and H5N4) analyzed by PGC-LC-MS (PDF)
**Table S3,** overview of the human plasma glycoforms detected by the CE-ESI-MS platform (XLSX)

## References

[ref1] Everest-Dass A. V.; Moh E. S. X.; Ashwood C.; Shathili A. M. M.; Packer N. H. Human Disease Glycomics: Technology Advances Enabling Protein Glycosylation Analysis – Part 1. Expert Rev. Proteomics 2018, 15, 165–182. 10.1080/14789450.2018.1421946. 29285957

[ref2] Hu M.; Lan Y.; Lu A.; Ma X.; Zhang L.; Zhang L. Glycan-Based Biomarkers for Diagnosis of Cancers and Other Diseases: Past, Present, and Future. Prog. Mol. Biol. Transl. Sci. 2019, 162, 1–24. 10.1016/bs.pmbts.2018.12.002. 30905444

[ref3] Varki A. Glycan-Based Interactions Involving Vertebrate Sialic-Acid-Recognizing Proteins. Nature 2007, 446, 1023–1029. 10.1038/nature05816. 17460663

[ref4] Anthony R. M.; Nimmerjahn F.; Ashline D. J.; Reinhold V. N.; Paulson J. C.; Ravetch J. V. Recapitulation of IVIg Anti-Inflammatory Activity with a Recombinant IgG Fc. Science 2008, 320, 373–376. 10.1126/science.1154315. 18420934 PMC2409116

[ref5] Vreeker G. C. M.; Wuhrer M. Reversed-Phase Separation Methods for Glycan Analysis. Anal. Bioanal. Chem. 2017, 409, 359–378. 10.1007/s00216-016-0073-0. 27888305 PMC5203856

[ref6] Cao W.-Q.; Liu M.-Q.; Kong S.-Y.; Wu M.-X.; Huang Z.-Z.; Yang P.-Y. Novel Methods in Glycomics: A 2019 Update. Expert Rev. Proteomics 2020, 17, 11–25. 10.1080/14789450.2020.1708199. 31914820

[ref7] Cheng M.; Shu H.; Yang M.; Yan G.; Zhang L.; Wang L.; Wang W.; Lu H. Fast Discrimination of Sialylated *N*-Glycan Linkage Isomers with One-Step Derivatization by Microfluidic Capillary Electrophoresis–Mass Spectrometry. Anal. Chem. 2022, 94, 4666–4676. 10.1021/acs.analchem.1c04760. 35258917

[ref8] Melmer M.; Stangler T.; Premstaller A.; Lindner W. Comparison of Hydrophilic-Interaction, Reversed-Phase and Porous Graphitic Carbon Chromatography for Glycan Analysis. J. Chromatogr. A 2011, 1218, 118–123. 10.1016/j.chroma.2010.10.122. 21122866

[ref9] Lu G.; Crihfield C. L.; Gattu S.; Veltri L. M.; Holland L. A. Capillary Electrophoresis Separations of Glycans. Chem. Rev. 2018, 118, 7867–7885. 10.1021/acs.chemrev.7b00669. 29528644 PMC6135675

[ref10] Schwedler C.; Kaup M.; Weiz S.; Hoppe M.; Braicu E. I.; Sehouli J.; Hoppe B.; Tauber R.; Berger M.; Blanchard V. Identification of 34 *N*-Glycan Isomers in Human Serum by Capillary Electrophoresis Coupled with Laser-Induced Fluorescence Allows Improving Glycan Biomarker Discovery. Anal. Bioanal. Chem. 2014, 406, 7185–7193. 10.1007/s00216-014-8168-y. 25234305

[ref11] Sanchez-Lopez E.; Kammeijer G. S. M.; Crego A. L.; Marina M. L.; Ramautar R.; Peters D. J. M.; Mayboroda O. A. Sheathless CE-MS Based Metabolic Profiling of Kidney Tissue Section Samples from a Mouse Model of Polycystic Kidney Disease. Sci. Rep. 2019, 9, 806 10.1038/s41598-018-37512-8. 30692602 PMC6349881

[ref12] Schultz C. L.; Moini M. Analysis of Underivatized Amino Acids and Their D/L-Enantiomers by Sheathless Capillary Electrophoresis/Electrospray Ionization Mass Spectrometry. Anal. Chem. 2003, 75, 1508–1513. 10.1021/ac0263925. 12659216

[ref13] Lageveen-Kammeijer G. S. M.; de Haan N.; Mohaupt P.; Wagt S.; Filius M.; Nouta J.; Falck D.; Wuhrer M. Highly Sensitive CE-ESI-MS Analysis of *N*-Glycans from Complex Biological Samples. Nat. Commun. 2019, 10, 2137 10.1038/s41467-019-09910-7. 31086181 PMC6513864

[ref14] Busnel J.-M.; Schoenmaker B.; Ramautar R.; Carrasco-Pancorbo A.; Ratnayake C.; Feitelson J. S.; Chapman J. D.; Deelder A. M.; Mayboroda O. A. High Capacity Capillary Electrophoresis-Electrospray Ionization Mass Spectrometry: Coupling a Porous Sheathless Interface with Transient-Isotachophoresis. Anal. Chem. 2010, 82, 9476–9483. 10.1021/ac102159d. 21028888

[ref15] Heemskerk A. A. M. Exploring the Proteome by CE-ESI-MS. Doctoral thesis, Leiden University, 2016. https://openaccess.leidenuniv.nl/handle/1887/38868.

[ref21] Kammeijer G. S. M.; Kohler I.; Jansen B. C.; Hensbergen P. J.; Mayboroda O. A.; Falck D.; Wuhrer M. Dopant Enriched Nitrogen Gas Combined with Sheathless Capillary Electrophoresis–Electrospray Ionization-Mass Spectrometry for Improved Sensitivity and Repeatability in Glycopeptide Analysis. Anal. Chem. 2016, 88, 5849–5856. 10.1021/acs.analchem.6b00479. 27119460

[ref16] Palmisano G.; Larsen M. R.; Packer N. H.; Thaysen-Andersen M. Structural Analysis of Glycoprotein Sialylation – Part II: LC-MS Based Detection. RSC Adv. 2013, 3, 22706–22726. 10.1039/c3ra42969e.

[ref17] Green E. D.; Adelt G.; Baenziger J. U.; Wilson S.; Van Halbeek H. The Asparagine-Linked Oligosaccharides on Bovine Fetuin. Structural Analysis of *N*-Glycanase-Released Oligosaccharides by 500-Megahertz 1H NMR Spectroscopy. J. Biol. Chem. 1988, 263, 18253–18268. 10.1016/S0021-9258(19)81354-6. 2461366

[ref18] Lane C. S.; McManus K.; Widdowson P.; Flowers S. A.; Powell G.; Anderson I.; Campbell J. L. Separation of Sialylated Glycan Isomers by Differential Mobility Spectrometry. Anal. Chem. 2019, 91, 9916–9924. 10.1021/acs.analchem.9b01595. 31283185 PMC6686149

[ref19] Zhou S.; Huang Y.; Dong X.; Peng W.; Veillon L.; Kitagawa D. A. S.; Aquino A. J. A.; Mechref Y. Isomeric Separation of Permethylated Glycans by Porous Graphitic Carbon (PGC)-LC-MS/MS at High Temperatures. Anal. Chem. 2017, 89, 6590–6597. 10.1021/acs.analchem.7b00747. 28475308 PMC5761069

