# Supplementary material for: N-Glycan Isomer Differentiation by Zero Flow Capillary Electrophoresis Coupled to Mass Spectrometry
Source: Anal Chem. 2022 Sep 13;94(38):12954–9. doi: 10.1021/acs.analchem.2c02840 (PMC9523619; doi:10.1021/acs.analchem.2c02840)
Supplement: Supplementary file 1 — ac2c02840_si_001.pdf [file ac2c02840_si_001.pdf]

Supporting Information I: Experimental Section, Figures and Tables

N-glycan isomer differentiation by zero flow capillary electrophoresis coupled to mass spectrometry

Sander Wagt[1,‡], Noortje de Haan[1,†,‡], Wenjun Wang[1], Tao Zhang[1], Manfred Wuhrer[1], Guinevere S.M. Lageveen-Kammeijer[1*]

1 Leiden University Medical Center, Center for Proteomics and Metabolomics, 2300 RC Leiden, The Netherlands

† Current address: University of Copenhagen, Copenhagen Center for Glycomics, 2200 Copenhagen, Denmark

‡ These authors contributed equally: Sander Wagt and Noortje de Haan.

* Correspondence: Guinevere S.M. Lageveen-Kammeijer, Leiden University Medical Center, Center for Proteomics and Metabolomics, P.O. Box 9600, 2300 RC Leiden, The Netherlands; g.s.m.kammeijer@lumc.nl; Tel: +31-71-52-69384

TABLE OF CONTENTS

S-1: Experimental Section **2**

Chemicals 2

N-glycan sample preparation for CE-ESI-MS measurements 2

N-glycan sample preparation for PGC-nano-LC-MS measurements 2

Dynamically coated neutral capillary 2

PGC-nano-LC-MS/MS analysis 3

PGC-nano-LC-MS/MS data processing 3

S-2: Supporting Figures **4**

Figure S1, CE-ESI-MS analysis of released and positively labelled *N*-glycans on different neutral capillaries compared based on the separation efficiency. 4

Figure S2, Relative peak areas obtained from the CE-ESI-MS analysis of derivatized and positively labelled *N*-glycans released from human plasma on a static neutrally coated capillary. 5

Figure S3, CE separation and structural elucidation of isomeric *N*-glycans with the composition H6N5S3 after differential sialic acid derivatization and positive reducing end labelling. 6

S-3: Supporting Tables **7**

Table S1: Schematic overview of method properties per capillary type 7

Table S2, Sensitivity assessment (*N* = 3) using two glycan standards (H3N4 and H5N4) analyzed by PGC-LC-MS 7

References **7**

S-1: EXPERIMENTAL SECTION

Chemicals

LC-MS grade water (cat nr. 15641400) was obtained from Riedel-de Haën (Buchs, Switzerland). Acetonitrile (MeCN; cat. nr. 1203502) was obtained from Biosolve (Valkenswaard, The Netherlands). Ammonium bicarbonate (ABC; cat. nr. 09830), glacial acetic acid (HAc; cat. nr. 15625660), 1-hydroxybenzotriazole (HOBt; cat. nr. 54802) hydrate, dimethyl sulfoxide (DMSO; cat. nr. D8418), Nonidet P-40 substitute (NP-40; cat. nr. M158)), 50% sodium hydroxide (NaOH; cat. nr. 71686), 40% dimethylamine (cat nr. 426458), 28% ammonium hydroxide (cat. nr. 221228), hydrochloric acid solutions of 1 M (HCl; cat. nr. 15653530) and 37% (HCl; cat. nr. 258148, Dowex cation-exchange resin (50W-X8; cat. nr. 217492), trifluoroacetic acid (TFA; cat. nr. 1.08178.0050), fetuin from fetal bovine serum (FBS; cat. nr. F3385), DL-dithiothreitol (DTT; cat. nr. D0632), sodium chloride (NaCl; cat. nr. 1.06404.1000), sodium borohydride ($NaBH_4$; cat. nr. 452882), and 7.5 M ammonium acetate (AmAc; cat. nr. A2706) were purchased from Sigma-Aldrich (Steinheim, Germany). Potassium hydroxide (KOH; cat. nr. P1767) was purchased from Honeywell Fluka. Solid phase extraction (SPE) bulk sorbent Carbograph (cat. nr. 5122145) was obtained from Grace Discovery sciences (Columbia, MD). Trifluoroacetic acid (TFA; cat. nr. 1.08178.0050), methanol (MeOH; cat. nr. 1.06009.2500), 2-propanol (cat. nr. 34965), ethanol (EtOH; cat. Nr 1.00983.1000), sodium dodecyl sulphate (SDS; cat. nr. L3771), disodium hydrogen phosphate dihydrate ($Na_2HPO_4 \cdot H_2O$; cat. nr. 1197530250) and potassium dihydrogen phosphate (KH_2PO_4; cat. nr. 1048730250) were acquired from Merck (Darmstadt, Germany). Milli-Q water (MQ) was obtained with a Q-Gard 2 system (Millipore, Amsterdam, Netherlands) and 1-ethyl-3-(3-dimethylaminopropyl)carbodiimide hydrochloride (EDC; cat. nr. 24810) was purchased from Fluorochem (Hadfield, United Kingdom). Ultratrol dynamic pre-coat LN was obtained from Target Discovery (Palo Alto, CA), 1-(hydrazinocarbonylmethyl)pyridinium chloride (Girard's reagent P.; GirP; cat. nr.G0030) from TCI Development Co. Ltd. (Tokyo, Japan) and peptide-*N*-glycosidase F (PNGase F; cat. nr. 11365177001) from Roche Diagnostics (Mannheim, Germany). Phosphate-buffered saline (10x PBS) was prepared in-house, containing 57 g/L $Na_2HPO_4 \cdot 2H_2O$, 5 g/L KH_2PO_4, and 85 g/L NaCl. H3N4, H5N4, and H7N6 (CN-NGA2-10U, CN-NA2-10U, CN-NA4-10U, respectively) were kindly provided by Ludger Ltd. (Abingdon, UK).

N-glycan sample preparation for CE-ESI-MS measurements

The *N*-glycan sample was prepared according to the recently described workflow,[1] consisting of an *N*-glycan release, linkage-specific sialic acid derivatization, cotton hydrophilic interaction liquid chromatography solid-phase extraction (HILIC SPE) and reducing end labeling with the positively charged GirP, with some minor modifications. Briefly, *N*-glycans were released from 100 µL of human plasma or 10 µL of fetuin by adding 200 µL 2% SDS or 20 µL 2% SDS and incubating for 10 min at 60 °C, respectively. Subsequently, 200 µL (human plasma) or 20 µL (fetuin) of release mixture (5 mU PNGase F in 2% NP-40 and 2.5x PBS) was added and the samples were incubated overnight at 37 °C. Linkage-specific sialic acid derivatization through ethyl esterification and ammonia amidation was performed by mixing 1 µL of the released *N*-glycans with 20 µL of 250 mM EDC and 250 mM HOBt in EtOH, followed by an incubation of 1 h at 37 °C. Then, 4 µL 28% ammonium hydroxide solution was added, followed by 2 h incubation at 37 °C. Prior to the *N*-glycan purification by cotton HILIC SPE, 24 µL MeCN was added to the reaction mixture. Purified *N*-glycans were eluted in 10 µL MQ. For the reducing end labeling with GirP, 5 µL of derivatized and purified glycans were dried in the vacuum concentrator for 15 min at 60 °C and reconstituted in 2 µL 50 mM GirP in 10% HAc/90% EtOH (*v/v*), which was shaken on a shaking plate for 5 min and subsequently incubated for 2 h at 60 °C. Finally, the sample was dried by vacuum concentration or purified by cotton HILIC SPE as described above.

N-glycan sample preparation for PGC-nano-LC-MS measurements

The *N*-glycan standards H3N4, H5N4 and H7N6 were prepared in H_2O at different concentrations of 10 pM, 1 pM, 500 fM, 100 fM, 50 fM, and 10 fM. Next, the *N*-glycan standards were reduced and purified as described previously.[2] In brief, the *N*-glycan standards were reduced to alditols in 30 µL of sodium borohydride (500 mM) in potassium hydroxide (50 mM) for 3 h at 50 °C. After desalting, the glycan alditols were collected by combining the flow-through and eluate, followed by a drying step using an Eppendorf Concentrator at 30 °C. A PGC SPE clean-up was performed to further purify the samples. Samples were dried in a SpeedVac concentrator directly and re-dissolved in 10 µL of water prior to PGC nano-LC-ESI-MS/MS analysis.

Dynamically coated neutral capillary

The dynamically coated neutral capillary, OptiMS silica surface cartridges (SCIEX) were coated with UT according to the recently described procedure[1]. In short, the bare-fused silica capillaries were conditioned according to the manufacturer's instructions. Afterwards, the separation line was coated by sequential 10 min rinsing steps with MeOH, H_2O, 0.1 M HCl and H_2O, followed by 15 min rinsing steps with 1 M NaOH and H_2O, after which the capillary was rinsed for 30 min with UT and then flushed for 30 min with BGE, all at 29 psi. Prior to each analysis the capillary was rinsed with 1 M NaOH (3 min), H_2O (4 min), UT (4 min) and BGE (3 min) using 29 psi. This was followed by an additional rinsing step with BGE (6 min, 100 psi), which removes any residual UT polymer from the spray tip and is essential to make the procedure compatible with MS detection. The separation was carried out using 20 kV, without applying any pressure.

PGC-nano-LC-ESI-MS/MS analysis

The analysis of *N*-glycans was performed on an Ultimate 3000 UHPLC system (Dionex/Thermo) equipped with a Hypercarb PGC trap column (5 µm Hypercarb Kappa, 320 µm x 30 mm, Thermo Fisher Scientific, Waltham, MA) and a Hypercarb PGC nano-column (3 µm Hypercarb 75 µm x 100 mm, Thermo Fisher Scientific) coupled to an amaZon ETD speed ion trap (Bruker Daltonics). Mobile phase A consisted of 10 mM ABC, while mobile phase B was 60% MeCN/10 mM ABC (*v/v*). For the analysis, 1 µL of the *N*-glycan standard mixture was injected and trapped on the trap column using a 6 µL/min loading flow in 2% buffer B for 5 min. Separation was achieved with a multi-step gradient of B: 2-9% in 1 min and 9-49% in 80 min, followed by a 10 min wash step using 95% of B, all at a flow of rate of 0.6 µL/min and a column temperature of 45 °C. Ionization was achieved using the CaptiveSpray nanoBooster source (Bruker Daltonics) with isopropanol as dopant and a capillary voltage of 1000 V. The dry gas temperature was set at 280°C with a flow of 5 L/min and the nebulizer set at 3 psi. MS spectra were acquired within a *m/z* range of 500-1850 in enhanced mode, smart parameter setting (SPS) was set to *m/z* 1200, ion charge control (ICC) to 4 x 10^3 and maximum acquisition time of 200 ms. MS/MS spectra were generated using collision-induced dissociation over a *m/z* range from 100 to 2500 on the top three most abundant precursors, applying an isolation width of 3 Th. The fragmentation cut-off was set to 27% with 100% fragmentation amplitude using the Enhanced SmartFrag option from 30-120% in 32 ms and ICC was set to 150,000.

PGC-nano-LC-MS Data processing

The PGC-LC-MS data was processed using DataAnalysis 5.0 (Build 203, Bruker Daltonics).

S-2: SUPPORTING FIGURES

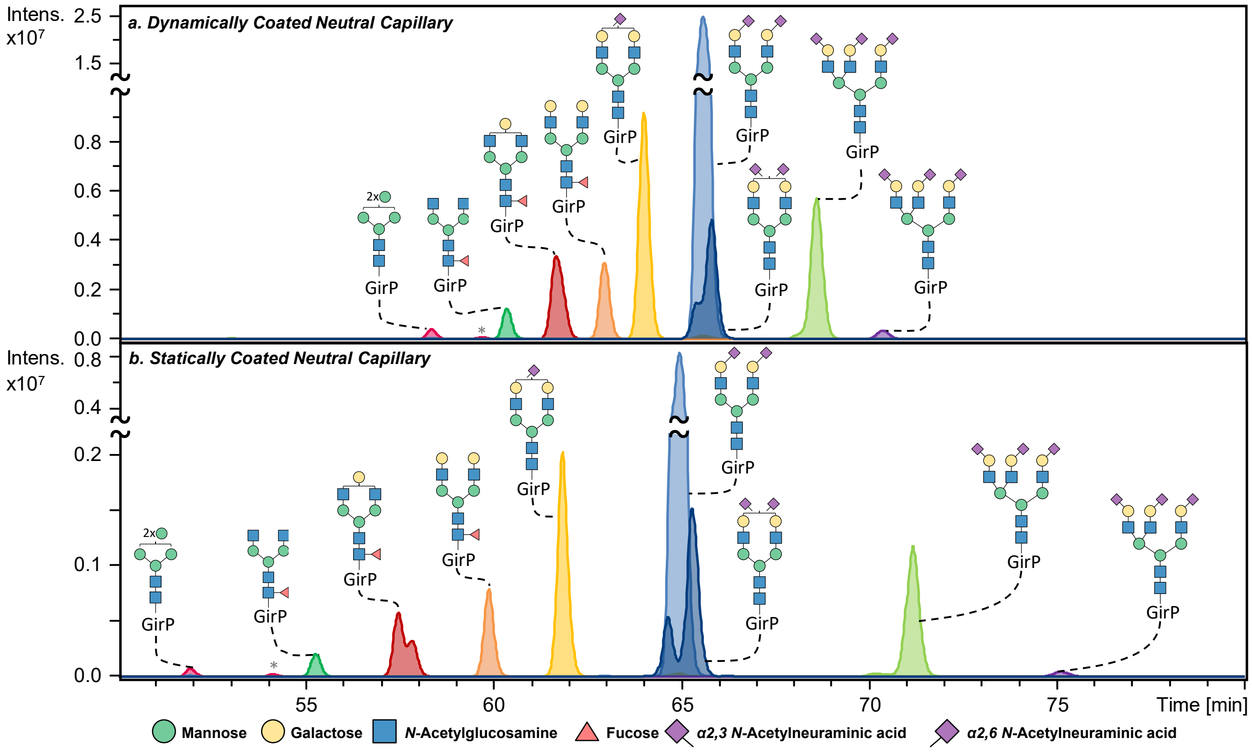

Supporting Figure S1: CE-ESI-MS analysis of released and positively labelled *N*-glycans on different neutral capillaries compared based on the separation efficiency. Extracted ion electropherograms (EIEs) from nine of the most abundant *N*-glycans observed across the entire migration window, separated on an **(a)** in-house dynamically coated neutral capillary compared to **(b)** a commercially available statically coated neutral capillary (OptiMS, SCIEX). *****: in-source decay product corresponding to the *N*-glycan structure assigned to the colored trace as a result of the glycosidic bond cleavage from the precursor ion**.** The depicted structures are based on literature and knowledge on the biosynthetic pathway, where the monosaccharide linkages were not determined, except for the sialic acids**.**

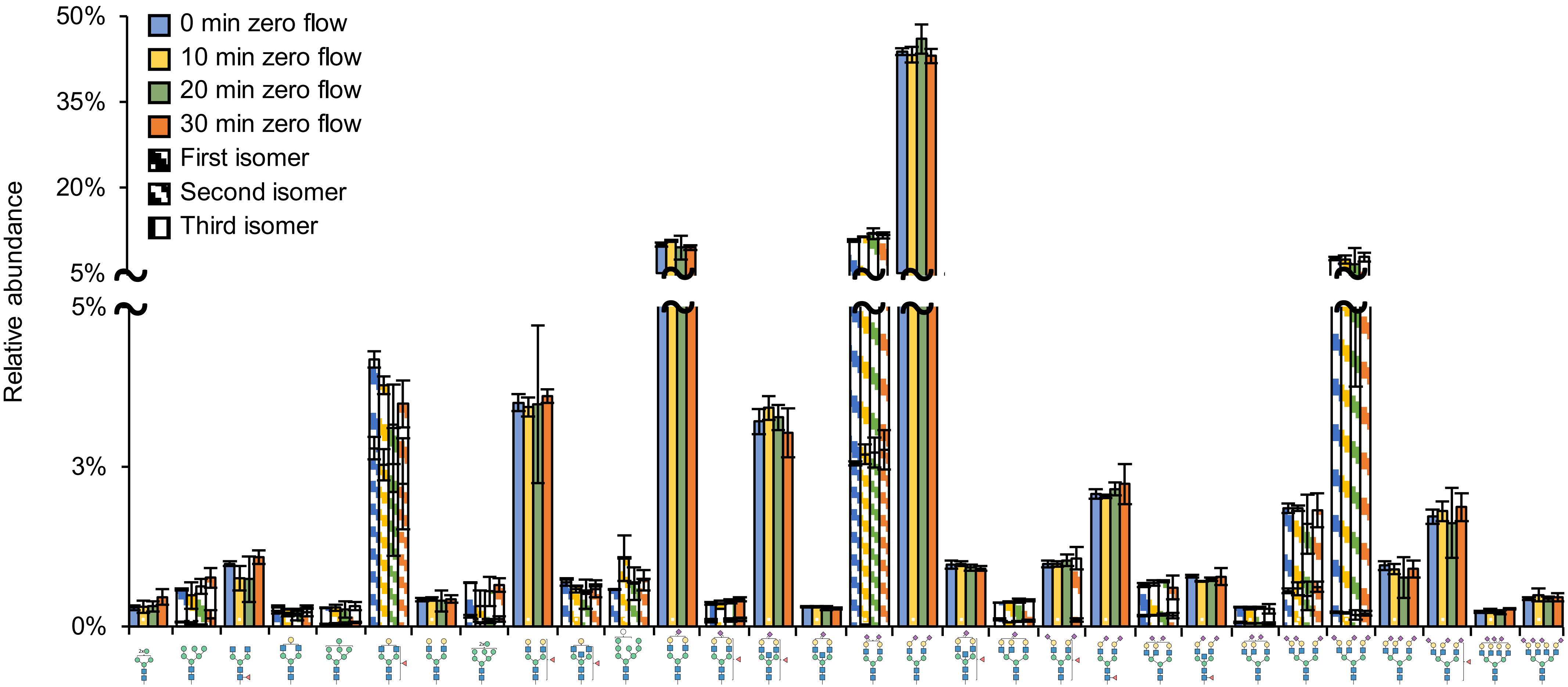

Supporting Figure S2: Relative peak areas obtained from the CE-ESI-MS analysis of derivatized and positively labelled *N*-glycans released from human plasma on a static neutrally coated capillary. The "zero flow" approach was applied for (**blue**) 0 min**,** (**yellow**) 10 min, (**green**) 20 min and (**orange**) 30 min. Pattern indicates whether isomers could be observed at a specific condition. Error bars represent the standard deviation (*N=3*).

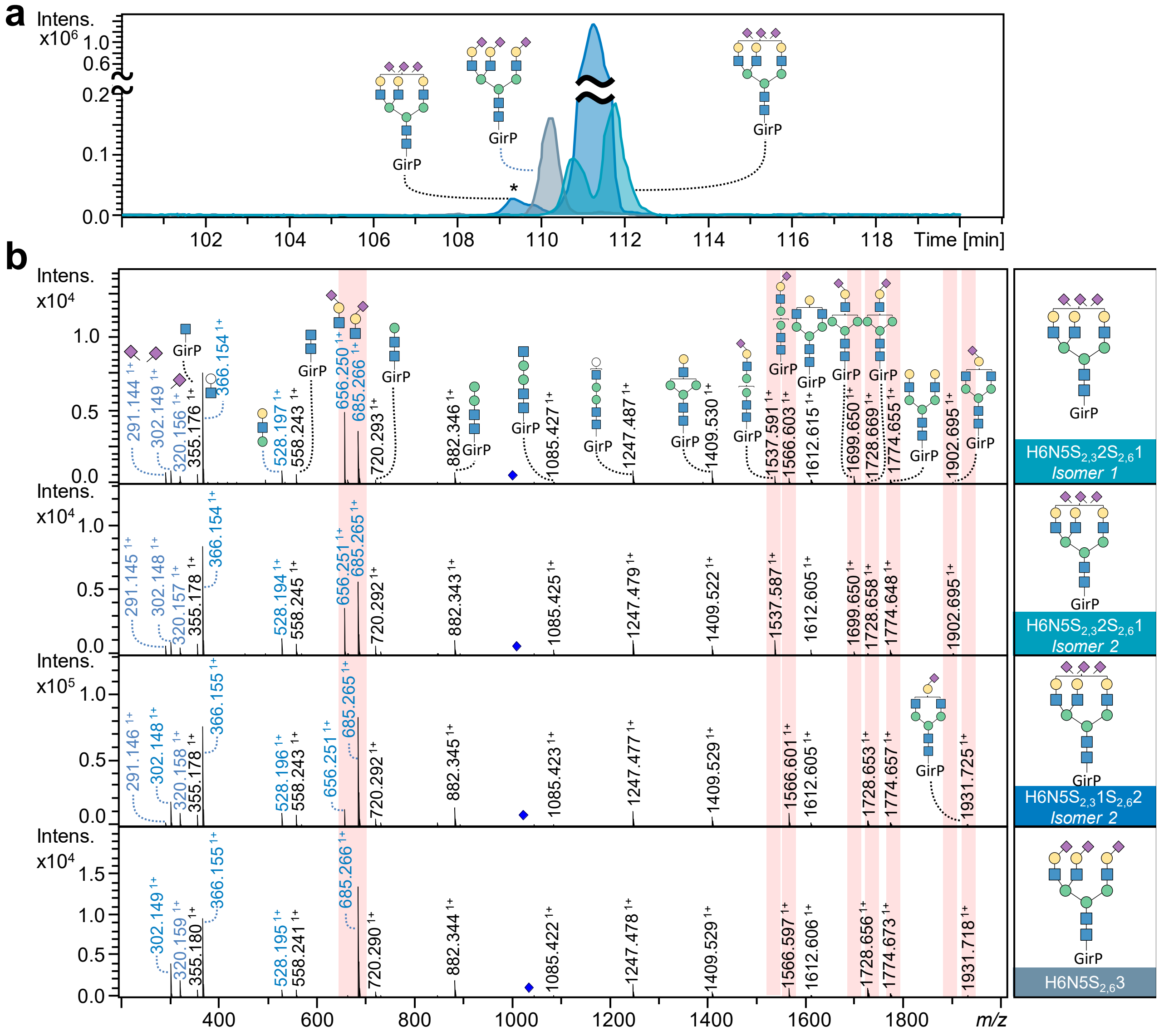

Supporting Figure S3: CE separation and structural elucidation of isomeric *N*-glycans with the composition H6N5S3 after differential sialic acid derivatization and positive reducing end labelling. (a) Extracted ion electropherograms of masses belonging to *N*-glycans H6N5S$_{2,3}$2S$_{2,6}$1, H6N5S$_{2,3}$1S$_{2,6}$2 and H6N5S$_{2,6}$3. **(b)** MS/MS fragmentation spectra obtained from the peaks in **(a)**. B- and Y-ions are labeled in blue and black, respectively. Highlight in red are the Y-ions of diagnostic value. * No fragmentation data available of the first isomer of H6N5S$_{2,3}$1S$_{2,6}$2 due to low intensity. Legend as in Supporting Figure S1.

S-3: SUPPORTING TABLES

Supporting Table S1: Schematic overview of method properties per capillary type. The separation power of the CE is improved in the zero flow step using the statically coated neutral capillary as the analytes have more time to separate solely on their electrophoretic mobility in the absence of EOF and hydrodynamic flow.

Capillary type	Step	EOF	Applied pressure	Electrophoretic mobility	ESI spray
**Bare-fused** [1]	NA	+	-	+	+
**Dynamically coated neutral**	NA	+	-	+	+
**Statically coated neutral**	(1) Zero flow	-	-	+	-
	(2) Applied pressure	-	+	+	+

Supporting Table S2: Sensitivity assessment (*N* = 3) using two glycan standards (H3N4 and H5N4) analyzed by PGC-LC-MS. The light-yellow color indicates the observed LOD for this analytical platform.

Dilution factor	Amount in reaction	Concentration after reduction (in 10 µL)	Amount consumed for PGC-LC-MS measurement (1 µL)	Amount consumed for PGC-LC-MS measurement (2 µL)	Amount consumed for PGC-LC-MS measurement (3 µL)
1	10000 fmol	1000 fmol/µL	1000 fmol	2000 fmol	3000 fmol
10	1000 fmol	100 fmol/µL	100 fmol	200 fmol	300 fmol
20	500 fmol	50 fmol/µL	50 fmol	100 fmol	150 fmol
100	100 fmol	10 fmol/µL	10 fmol	20 fmol	30 fmol
200	50 fmol	5 fmol/µL	5 fmol	10 fmol	15 fmol
1000	10 fmol	1 fmol/µL	1 fmol	2 fmol	3 fmol
2000	5 fmol	0.5 fmol/µL	0.5 fmol	1 fmol	1.5 fmol
10000	1 fmol	0.1 fmol/µL	0.1 fmol	0.2 fmol	0.3 fmol

REFERENCES

(1) Lageveen-Kammeijer, G.S.M., *et al. Highly sensitive CE-ESI-MS analysis of N-glycans from complex biological samples*, *Nat. Comm.*, *10*, 2137, **(2019)**.

(2) Zhang, T., *et al. Development of a 96-well plate sample preparation method for integrated N- and O-glycomics using porous graphitized carbon liquid chromatography-mass spectrometry*, *Mol. Omics*, *16*, 355-363, **(2020)**.
